# Knowledge, competencies and attitudes regarding external post-mortem physical examination: A survey among German post-graduate trainees in general practice

**DOI:** 10.1080/13814788.2017.1389884

**Published:** 2017-11-23

**Authors:** Jan Valentini, Katja Goetz, Kathrin Yen, Joachim Szecsenyi, Andrea Dettling, Stefanie Joos, Jost Steinhaeuser, Elisabeth Flum

**Affiliations:** ^a^ Institute of General Practice and Multi-Disciplinary Healthcare, University Hospital and Faculty of Medicine Tübingen Tübingen Germany; ^b^ Department of General Practice and Health Services Research, University of Heidelberg Hospital Heidelberg Germany; ^c^ Institute of Family Medicine, University Hospital Lübeck Germany; ^d^ Institute for Forensic and Traffic Medicine, University of Heidelberg Hospital Heidelberg Germany; ^e^ Health Department, City Mannheim Mannheim Germany

**Keywords:** General practitioner, legal medicine, medical education, post-graduate training, post-mortem examination

## Abstract

**Background:** The external post-mortem examination (EPME) is an important medical, legal and socio-economic task with far-reaching relevance; however, due to discrepancies between findings from EPMEs and actual cause of death, improvements in accuracy and quality are needed.

**Objectives:** To investigate knowledge, competencies and attitudes regarding EPME in general practitioner (GP) post-graduate trainees.

**Methods:** Before four post-graduate training courses on the EPME for general practitioner trainees, organized in 2014 in the German federal state of Baden-Wuerttemberg, a questionnaire on the EPME was distributed by the lecturer, completed by the GP post-graduate trainees and returned to the lecturer. The questionnaire consisted of 19 items related to three main categories: knowledge, competencies and attitudes.

**Results:** Out of 380 GP post-graduate trainees, 128 completed and returned the questionnaire (response rate 33.7%). Less than 18% felt adequately confident in identifying a natural cause of death and less than 5% felt adequately confident in identifying an unnatural cause of death. Only 33% consistently fully uncover the corpse for the EPME.

**Conclusion:** We found an important uncertainty in GP post-graduate trainees regarding their EPME knowledge and competencies.

KEY MESSAGES

- Of the GP post-graduate trainees, 66% perceived their knowledge regarding the procedure of the external post-mortem examination to be inadequate or mediocre.
- Only 3% of the GP post-graduate trainees feel ‘adequately’ confident to determine cause of death.
- External post-mortem examination courses are highly relevant to post-graduate training programmes and should be implemented.

## Introduction

The external post-mortem examination (EPME) plays an important medical, legal and socio-economic role. By EPME, we mean the medical duty by which the licensed physician verifies death, completes the medical certificate of cause of death, and is distinct to the ‘post-mortem’ autopsy used in English vernacular. In many European countries, the performance of the EPME and the completion of the death certificate is a medical duty for a general practitioner (GP), as for almost every licensed physician [1–3]. The medical task consists of examining for certain signs of death, thus verifying death. Further aspects are the estimation of the time of death and identification of the likely cause and manner of death, which are typically required on the medical certificate. In verifying death, the physician needs to differentiate between natural causes of death and unnatural causes of death (e.g. accidents or suicides) and plays an important legal role. Legal aspects may include revealing medical error or malpractice. At the socio-economic level, information documented on the medical certificate of cause of death can be used for insurance queries e.g. life insurance policies. Additionally, the EPME provides data on infectious disease aspects and epidemic monitoring [4,5].

Every physician in Germany, including GPs, is obliged by law to perform an EPME and to complete the medical certificate of cause of death. Information documented on the medical certificate of death is used for the national ‘cause of death’ statistics. This usage may have far-reaching implications for the regulation and strategy of the national healthcare system. In the case of an unnatural or unexplained cause of death, the police must be notified. Depending on the circumstances, criminal investigation will lead to further investigations e.g. an autopsy by a forensic doctor [6].

Despite the importance of these tasks, several publications have highlighted that the quality of the conduction of the EPME, as well as the completion of the death certificates, is unsatisfactory [4,5,7–9]. In Germany, some studies have reported a substantial discrepancy between the cause of death proposed in the EPME and the cause of death established in the autopsy [10,11]. Autopsies performed by a forensic pathologist are conducted in less than 5% of all death cases, which is low in comparison with international figures [12,13]. Results from autopsy studies suggest that an unnatural cause of death occurs in 33% to 50% of all cases of death and is more frequent than indicated in national death statistics [13–15]. Similar discrepancies between the cause of death suspected at the EPME and the cause of death identified in the autopsy are also reported internationally [16–19]. The reasons for these undetected cases are broad. They could range from aspects such as ‘out-of-hours’ service, poor lighting, distressed relatives or time pressure. However, the literature suggests that the main cause may be due to inadequate knowledge and performance of the examining doctors, who are typically not sufficiently trained in forensic medicine [15,20]. Recent studies suggest that practical training positively affects the accuracy and quality of the EPME [6,21–23]. This is of heightened relevance if the doctors themselves feel inadequately prepared for the responsibility asked of them.

The aim of this study was to gain an insight into the attitudes and self-perceived levels of knowledge, competencies and attitudes in EPME among GP post-graduate trainees in Germany.

## Methods

### Participants and data collection

As part of the educational meetings of the Verbundweiterbildung*^plus^*Baden-Württemberg, a GP post-graduate training programme, workshops on the topic of EPME were held in Heidelberg, Germany, during four educational workshop courses in 2014 [24,25]. Before the EPME course, a questionnaire on EPME was distributed by the lecturer, completed by the GP post-graduate trainees and returned to the lecturer. Participation in the course was voluntary and the data collection was anonymous; therefore, it was not possible to identify the participants. The questionnaire was developed based on a literature review and the work experiences of two authors (EF, JSt), who are GPs and are working in post-graduate general practitioner training.

### Questionnaire

The questionnaire consisted of 19 items related to three main categories: knowledge, competencies and attitudes. Table 2 shows the complete questionnaire.

**Table 2.** Questionnaire: Knowledge, competencies and attitudes of GP post-graduate trainees on external post-mortem physical examination (*n* = 128). The *n* varies due to lack of response.

	Adequate	Half-and-half	Inadequate
*(A) Knowledge*			
How would you estimate your knowledge generally on the topic of external post-mortem examination? (*n* = 127)	30.7%	54.3%	15.0%
How would you assess your knowledge on the specific procedure of the external post-mortem examination? (*n* = 128)	33.6%	52.3%	14.1%
	Yes	No	
Are you aware that the cause of death statistics in Germany relies on the information in the death certificates? (*n* = 127)	78.0%	22.0%	
*(B) Competencies*			
	Yes	No	
Do you always perform the external post-mortem examination on the fully uncovered corpse? (*n* = 118)	33.1%	66.9%	
	Adequate	Half-and-half	Inadequate
How confident are you in the assessment of whether you should call the police or not? (*n* = 127)	20.5%	55.1%	24.4%
How confident do you feel with the formal criteria required when filling out the death certificate? (*n* = 127)	32.3%	55.1%	12.6%
How confident do you feel in identifying the identification of certain signs of death? (*n* = 126)	61.1%	35.7%	3.2%
How confident do you feel in identifying a natural cause of death? (*n* = 124)	17.7%	74.2%	8.1%
How confident do you feel in identifying an unnatural cause of death? (*n* = 122)	4.9%	63.9%	31.1%
How confident do you feel in the judgment in situations where medical confidentiality may be broken? (*n* = 125)	7.2%	31.2%	61.6%
How confident do you feel in determining the cause of death? (*n* = 124)	3.2%	65.3%	31.5%
How confident do you feel in the documentation of the causal chain of cause of death? (*n* = 124)	6.5%	61.3%	32.3%
How confident do you feel in determining the time of death? (*n* = 122)	13.1%	46.7%	40.2%
How confident do you feel when specifying whether an infectious disease (as defined by the Infection Protection Act) is present? (*n* = 124)	12.1%	49.2%	38.7%
How confident do you feel in dealing with cases of death that have occurred in connection with a medical intervention? (*n* = 124)	3.2%	48.4%	48.4%
How confident do you feel in dealing with potential conflicts of interest, if you are at the same time the doctor in charge of the family of the deceased? (*n* = 119)	12.6%	59.7%	27.7%
*(C) Attitudes*			
	Positive	Neutral	Negative
What is your attitude towards the external post-mortem examination? (*n* = 128)	25.0%	67.2%	7.8%
	Yes	No	
To me, the external post-mortem examination means the last service of the physician for the patient (*n* = 125)	72.0%	28.0%	
Do you feel personally uncomfortable with the examination of the corpse? (*n* = 122)	35.2%	64.8%	

### Data analysis

The analysis was performed with SPSS version 22.0 (SPSS Inc., IBM, Armonk, NY, USA). Continuous data were summarized using means and standard deviations. The three-point Likert scale ratings were handled as categorical data and presented as frequency counts and percentages.

### Ethics

As stated by the ethical committee of the medical faculty of the University of Heidelberg and as declared in the German Federal Data Protection Act, ethical approval for an anonymous survey was not needed.

## Results

### Participants

Out of 380 GP post-graduate trainees who participated in the educational meetings, 128 trainees completed and returned the questionnaire (response rate 33.7%). Table 1 shows the demographic characteristics of the participants.

**Table 1.** Sociodemographic data: Description and characteristics of the study population (GP post-graduate trainees).

Characteristics^a^	Study population (*n* = 128)	
Age, mean (SD)	33.9 (6.7)	
Gender, *n* (%)		
Male	22 (18.0%)	
Training year, *n* (%)		
	1st	30 (24.2%)
	2nd	24 (19.4%)
	3rd	18 (14.5%)
	4th	26 (21.0%)
	5th	26 (21.0%)
Outpatient setting^b^, *n* (%)	67 (54.0%)	
Inpatient setting^c^, *n* (%)	57 (46.0%)	

^a^ The *n* varies due to lack of response.

^b^ Outpatient setting refers to GP trainees working with ambulatory patients in the community, e.g., in a GP practice.

^c^ Inpatient setting refers to GP trainees working with patients who have been admitted to the hospital, i.e., within the hospital.

The results are shown in Table 2, according to the three main categories of the questionnaire: knowledge, competencies and attitudes.

### Knowledge

Before the EPME course, 69% of the GP post-graduate trainees estimated that their knowledge on this topic was inadequate or mediocre. More than 66% of the participants stated in the self-assessment that their knowledge regarding the specific procedure of the external post-mortem examination is inadequate or mediocre.

### Competencies

Almost 67% of the GP post-graduate trainees claimed not to consistently, fully uncover the corpse before the EPME. Nearly 18% of the participants felt adequately confident in identifying a natural cause of death, fewer than 5% felt adequately confident in identifying an unnatural cause of death. Assuming that the participants were also the doctors responsible for the family of the deceased, fewer than 13% stated that they would feel adequately confident in dealing with a potential conflict of interest.

### Attitudes

Questions on attitudes showed that more than 35% felt personally uncomfortable with the examination of the corpse. Table 2 shows additional data and the complete questionnaire.

## Discussion

### Main findings

This study showed an important uncertainty in both theoretical and practical aspects of the EPME among GP post-graduate trainees in Germany. Three main findings: ‘identification of a natural or unnatural cause of death,’ ‘knowledge on practical conduction of post-mortem procedure,’ and ‘dealing with potential conflict of interest with family of deceased’ are discussed in detail below.

### Strengths and weaknesses

The main strength of the study is the valuable insight gained into the GP post-graduate trainees’ perception of their abilities regarding EPME. Importantly, they were an unselected cohort who performs EPME in their daily work.

When interpreting the results, some limitations should be considered. The items on the questionnaire were rated on a narrow three-point Likert scale ranging from one (adequate) to three (inadequate) and, as such, were susceptible to poorly differentiated data. For some questions, a large percentage of the participants answered the questions with two (mediocre). The questionnaires were more likely to have been reliably completed because the process was voluntary and anonymous. However, the sample is vulnerable to selection bias. The results of this study are therefore not generalizable because of the small sample size but they are nevertheless relevant for the discussion regarding improving quality in the EPME.

### Identification of a natural or unnatural cause of death

Identification of an unnatural or natural cause of death was self-assessed as adequate in only 5% to 18% of participants, respectively. The GP post-graduate trainees were evidently insecure concerning the determination of the cause of death given that almost 97% assessed their confidence as ‘inadequate’ or ‘mediocre’. These observations may be linked to the poor reliability and quality of EPME; something which has been severely criticized internationally and in Germany over the past decades [4,8,16,17].

### *Knowledge of the practical* *conduction of the post-mortem procedure*

According to recent literature knowledge on the practical conduction may be a prerequisite for quality improvement in the EPME [26,27]. In our study, it is remarkable that more than 86% of the GP post-graduate trainees stated that their knowledge on the specific procedure on the EPME is adequate or mediocre. However, in contradiction, merely 33% of the GP post-graduate trainees answered that they consistently fully uncover the corpse before performing the EPME. It remains unclear why this is the case. Similar results were also found by Vennemann et al., and Wilmes [3,27]. Although adequate procedural knowledge on the practical conduction of the EPME is considered essential, it can be assumed that the mere knowledge does not necessarily lead to an improved quality and reliability of the post-mortem examination [28]. Some studies indicate that more practical experience in EPME is needed to improve quality and accuracy [6,23]. Given the fact that an increment of practical experience is not always possible, e-learning tools could represent a valuable tool in medical education [29]. In 2010, the Institute of Legal Medicine of the University of Muenster developed and implemented an online tool for undergraduate medical education, which guides students to a complete EPME. The learning outcome was evaluated positively and a gain of confidence in conducting a real, EPME could be shown [30]. Permanent implementation of e-learning tools not only in undergraduate, but also in post-graduate training could, therefore, be considered.

### Dealing with a potential conflict of interest

Assuming that the participants were also the doctor responsible for the family of the deceased, more than 87% assessed their confidence as being either ‘inadequate’ or ‘mediocre’ with regard to dealing with a potential conflict of interest. This question is particularly relevant for the field of general practice because as family doctor in daily working routine such conflicts frequently arise. Especially in out-of-hospital deaths, the GP is often responsible for the external post-mortem examination and loyalties to the family could have an influence, e.g. obligation to maintain good relations, pressure to accelerate the process, life insurance queries, etc.

### Implications

Further investigations with longitudinal observational data are needed to establish as to whether the information gleaned from the courses translates into improved practice of the participants e.g., a lower error rate in EPME. Qualitative studies will also be relevant to understand how and why GP post-graduate trainees act the way they do and what their difficulties and ambiguities are.

Regular training in EPME should be intensified not only in undergraduate medical education but also be mandatory in post-graduate training. The obligatory implementation of courses on this topic in post-graduate training programmes could make a profound difference to future practice.

## Conclusion

This study suggests that there may be a major uncertainty regarding EPME among GP post-graduate trainees in Germany.

## References

[1] Parzeller M, Dettmeyer R, Bratzke H. External and internal post mortem examination according to the German code of criminal procedure (StPO)–review of section 87 StPO with special emphasis on the forensic autopsy. Arch Kriminol. 2009;223:1–23. 19323147

[2] Dettmeyer RVM. Ärztliche Leichenschau in Deutschland. Rechtsgrundlagen Rechtsmedizin. 2009;19:391–398.

[3] Wilmes SM. Die Praxis der ärztlichen Leichenschau im ambulanten Bereich in Hamburg [Dissertation]. Medizinische Fakultät der Universität Hamburg; 2015.

[4] Selinger CP, Ellis RA, Harrington MG. A good death certificate: improved performance by simple educational measures. Postgrad Med J. 2007;83:285–286. 17403959 10.1136/pgmj.2006.054833 PMC2600020

[5] Myers KA, Farquhar DR. Improving the accuracy of death certification. CMAJ. 1998;158:1317–1323. 9614825 PMC1229326

[6] Schroder AS, Wilmes S, Sehner S, et al Post-mortem external examination: competence, education and accuracy of general practitioners in a metropolitan area. Int J Legal Med. 2017;131:1701–1706. 28210814 10.1007/s00414-017-1559-9

[7] Große Perdekamp M, Pollak S, Bohnert M, et al Äußere Leichenschau. Rechtsmedizin. 2009;19:413–417.

[8] Madea B. Strukturelle Probleme bei der Leichenschau. Rechtsmedizin. 2009;19:399–406.

[9] Gleich S, Viehöver S, Stäbler P, et al Falsch bescheinigter natürlicher Tod nach ärztlicher Leichenschau. Rechtsmedizi. 2017;27:2–7.

[10] Modelmog D, Roland G. Der Stellenwert von Obduktionsergebnissen. Dtsch Arztebl. 1992;89:A 3434.

[11] Heide S, Stiller D, Hilbig F, et al Efficiency of inspections of the corpse before cremation performed in the area of the Halle University medical centre. Arch Kriminol. 2013;232:161–177. 24547618

[12] Madea B, Rothschild M. The post mortem external examination: determination of the cause and manner of death. Dtsch Arztebl Int. 2010;107:575–586. quiz 87–88. 20830284 10.3238/arztebl.2010.0575 PMC2936051

[13] Brinkmann B, Du Chesne A, Vennemann B. Recent data for frequency of autopsy in Germany. Dtsch Med Wochenschr. 2002;127:791–795. 11951136 10.1055/s-2002-25021

[14] Eckstein P, Schyma C, Madea B. Medicolegal experiences in external post-mortem examinations before cremation—a retrospective analysis of the years 1998–2008. Arch Kriminol. 2010;225:145–158. 20642253

[15] Brinkmann B, Banaschak S, Bratzke H, et al Errors in autopsy in Germany. Results of a multicenter study (I). Arch Kriminol. 1997;199:1–12. 9157831

[16] Roulson J, Benbow EW, Hasleton PS. Discrepancies between clinical and autopsy diagnosis and the value of post mortem histology; a meta-analysis and review. Histopathology. 2005;47:551–559. 16324191 10.1111/j.1365-2559.2005.02243.x

[17] Lorin de la Grandmaison G, Fermanian C, Durigon M. Analysis of discrepancies between external body examination and forensic autopsy. Am J Forensic Med Pathol. 2008;29:40–42. 19749615 10.1097/PAF.0b013e318165c77b

[18] Jackowski C, Hausmann R, Jositsch D. Eine Dunkelziffer bei Tötungsdelikten in der Schweiz. Kriminalistik. 2014;10:607–614.

[19] Minelli N, Marchetti D. Discrepancies in death certificates, public health registries, and judicial determinations in Italy. J Forensic Sci. 2013;58:705–710. 23488733 10.1111/1556-4029.12114

[20] Brinkmann B. Errors in autopsy in Germany. Results of a multicenter study (II). Arch Kriminol. 1997;199:65–74. 9235875

[21] Anders S, Mueller M, Sperhake JP, et al Autopsy in undergraduate medical education—what do students really learn? Int J Legal Med. 2014;128:1031–1038. 24487723 10.1007/s00414-014-0974-4

[22] Anders S, Fischer-Bruegge D, Fabian M, et al Teaching post-mortem external examination in undergraduate medical education—the formal and the informal curriculum. Forensic Sci Int. 2011;210:87–90. 21376489 10.1016/j.forsciint.2011.02.008

[23] Madea B, Bajanowski T, Peschel O, et al Kontinuierliche ärztliche Fortbildung zum Thema Leichenschau. Rechtsmedizin. 2011;21:51–54.

[24] Flum E, Magez J, Aluttis F, et al Das Schulungsprogramm der Verbundweiterbildungplus Baden-Württemberg: Entwicklung und Implikationen für die Implementierung von Verbundweiterbildungsprogrammen in Deutschland. Z Evid Fortbild Qual Gesundhwes. 2016;112:54–60. 27172786 10.1016/j.zefq.2016.03.012

[25] Steinhauser J, Roos M, Haberer K, et al Report from general practice: the composite graduate education(plus) program of the Baden-Wurttemberg general practice competence center—development, implementation and prospects. Z Evid Fortbild Qual Gesundhwes. 2011;105:105–109. 21496778 10.1016/j.zefq.2011.02.002

[26] Schwarz CS, Gutmann I, Bux R, et al Die Leichenschau. Dtsch Med Wochenschr. 2015;140:1148–1152. 26230067 10.1055/s-0041-103283

[27] Vennemann B, Du Chesne A, Brinkmann B. The practice of medical postmortem examination. Dtsch Med Wochenschr. 2001;126:712–716. 11446027 10.1055/s-2001-15033

[28] Frank JR, Snell L, Sherbino J, CanMEDS 2015 physician competency framework. Ottawa: The Royal College of Physicians and Surgeons of Canada; 2015.

[29] Smolle J. Virtual medical campus: the increasing importance of E-learning in medical education. GMS Z Med Ausbild. 2010;27:Doc29. 21818198 10.3205/zma000666 PMC3140364

[30] Schmeling A, Kellinghaus M, Becker JC, et al A web-based e-learning programme for training external post-mortem examination in curricular medical education. Int J Legal Med. 2011;125:857–861. 21901359 10.1007/s00414-011-0613-2

